# Treatment of Tooth Wear Using Direct or Indirect Restorations: A Systematic Review of Clinical Studies

**DOI:** 10.3390/bioengineering9080346

**Published:** 2022-07-27

**Authors:** Louis Hardan, Davide Mancino, Rim Bourgi, Carlos Enrique Cuevas-Suárez, Monika Lukomska-Szymanska, Maciej Zarow, Natalia Jakubowicz, Juan Eliezer Zamarripa-Calderón, Laura Kafa, Olivier Etienne, François Reitzer, Naji Kharouf, Youssef Haïkel

**Affiliations:** 1 Department of Restorative Dentistry, School of Dentistry, Saint-Joseph University, Beirut 11072180, Lebanon; louis.hardan@usj.edu.lb (L.H.); rim.bourgi@net.usj.edu.lb (R.B.); 2 Department of Biomaterials and Bioengineering, INSERM UMR_S 1121, Biomaterials and Bioengineering, 67000 Strasbourg, France; endodontiefrancaise@outlook.com (D.M.); olivier.etienne@unistra.fr (O.E.); f.reitzer@gmail.com (F.R.); youssef.haikel@unistra.fr (Y.H.); 3 Department of Endodontics, Faculty of Dental Medicine, Strasbourg University, 67000 Strasbourg, France; 4 Pôle de Médecine et Chrirugie Bucco-Dentaire, Hôpital Civil, Hôpitaux Universitaires de Strasbourg, 67000 Strasbourg, France; 5 Dental Materials Laboratory, Academic Area of Dentistry, Autonomous University of Hidalgo State, Circuito Ex Hacienda La Concepción S/N, San Agustín Tlaxiaca 42160, Mexico; eliezerz@uaeh.edu.mx; 6 Department of Restorative Dentistry, Medical University of Lodz, 251 Pomorska St., 92-213 Lodz, Poland; monika.lukomska-szymanska@umed.lodz.pl; 7 “NZOZ SPS Dentist” Dental Clinic and Postgraduate Course Centre, pl. Inwalidow 7/5, 30-033 Cracow, Poland; dentist@dentist.com.pl (M.Z.); nljakubowicz@gmail.com (N.J.); 8 Department of Oral Medicine, Faculty of Dentistry, Tishreen University, Lattakia 0100, Syria; laurakafa@gmail.com; 9 Department of Prosthetic, Faculty of Dental Medicine, Strasbourg University, 67000 Strasbourg, France

**Keywords:** crowns, fixed prosthodontics, resin composite, survival

## Abstract

Tooth wear is considered a well-developed issue in daily clinical practice; however, there is no standard protocol for treatment. The aim of this manuscript was to systematically review the literature to evaluate the clinical outcomes of direct or indirect restorations for treating tooth wear. A literature search was conducted through the PubMed MedLine, Scopus, ISI Web of Science, Scielo, and EMBASE databases up to 29 April 2022. Clinical studies evaluating the clinical performance of direct or indirect restorations for treating tooth wear for a minimum follow-up of 6 months were included in the review. A total of 2776 records were obtained from the search databases. After full-text reading, 16 studies were included in the qualitative analysis. Considering the high heterogenicity of the studies included, a meta-analysis could not be performed. All studies included the rehabilitation of anterior and posterior teeth with extensive wear, using both indirect and direct restorations for a maximum follow-up of 10 years. Restoration materials included ceramo-metal crowns, full gold crowns, lithium disilicate ceramic, zirconia, polymer infiltrated ceramic networks, and resin composites. Most of the reports assessed the survival rate of the restorations and the clinical features using the United States Public Health Service (USPHS) Evaluation System criteria. Contradictory discoveries were perceived concerning the type of restoration with better clinical performance. Considering the current literature available, there is no evidence in the superiority of any restoration technique to ensure the highest clinical performance for treating tooth wear.

## 1. Introduction

Currently, tooth wear, defined as a simple loss of dental substance, is considered a well-developed issue in daily clinical practice [1,2]. Progressive tooth wear with large zones of exposed dentinal surface is a restorative drawback for older patients who want to maintain their remaining dental structure. Usually, conventional restorative techniques for these patients include costly dental laboratory-fabricated crowns and fixed prostheses as a full-mouth rehabilitation [3].

In general, tooth wear, an irreversible non-carious loss of tooth structure, is an effect of diverse mechanisms, such as a dissolution by means of acidic substances of hard tissues (erosion), an interaction with exogenous materials (abrasion), or tooth-to-tooth contact (attrition) [4]. These mechanisms of tooth wear frequently act chronologically or in synchrony, which can enhance extreme tooth wear at a somewhat young age [5]. This circumstance creates plentiful problems, such as changes in vertical dimension of occlusion with possible functional deficiency, increased tooth hypersensitivity, pulp involvement, and perhaps diminished esthetic appearance [6]. Essentially, several factors, such as pain, speech, chewing ability, taste, and esthetics could affect aspects of patient quality of life [7]. The multiple factors of tooth wear and its associated restorative process are difficult for dental practitioners, as a multifaceted holistic rehabilitation program is needed that addresses changes in the occlusal surface [8].

Several approaches have been designated in the literature to rehabilitate a worn dentition by means of direct composite restorations [9,10,11,12,13,14], indirect restorations of lithium disilicate [15], composite resin [16], polymer infiltrated ceramic networks [17,18], and combined techniques [19,20,21]. However, the accessible clinical recommendations for any restorative method of worn dentition were quite limited, and a systematic review was not available on which techniques and materials are favored [22]. Most of the studies used direct or indirect resin composites to restore specifically worn anterior teeth and had stated failure rates of nearly 10% [16,23,24]. It is worth mentioning that failure rate, defined as the frequency at which a restoration fails, is exceptionally high when treating dental wear.

Preventive procedures and measures for advocating and examining tooth wear need to be in place before the initiation of any restorative procedure [12,25]. Worn dentition must be treated with a reversible, adhesive, additive method whenever achievable [26]. Nevertheless, patients frequently seek solutions when tooth wear has progressed significantly [27], and, in certain circumstances, prosthetic rehabilitation might be required.

The multifactorial assessment regarding severe tooth wear must be established based on its severity as well as the patient’s needs [28]. This assessment is not regularly done because the remaining tooth structure and the impact of persistent mechanical and chemical processes influences the performance of the restoration [7,29,30]. Because using composite resin could lead to a fracture and amplify the long lasting costs [16,31], a full crown persists as the chosen treatment [31], and metal–ceramic restorations are the average treatment for fixed partial dentures and crowns [32]. The drawback of metal–ceramic restorations is the grayish discoloration at the gingival margin. High-strength ceramic constituents such as lithium disilicate and zirconia have been developed and become popular due to their biocompatibility [33,34]. Compared to multilayer restorations, monolithic restorations are thinner, need less reduction of the tooth surface [35], and do not chip [36].

So far, there is no standard protocol for the treatment of individuals with tooth wear. Thus, the aim of this paper was to systematically review the literature to evaluate the clinical outcomes of direct or indirect restorations for treating tooth wear.

## 2. Materials and Methods

This review was implemented in agreement with the PRISMA 2020 instructions [37]. The registration protocol was carried out in Open Science Framework with the registration number 0000-0002-2759-8984. The following PICOS framework was used: population, dental substrate; intervention, indirect restorations; control, direct restorations; outcomes, Federation Dentaire Internationale (FDI) or United States Public Health Service (USPHS) criteria; and study design, clinical trials. The research question was: “What is the best treatment for treating tooth wear: direct or indirect restorations?”

### 2.1. Literature Search

A literature search was directed through the PubMed MedLine, Scopus, ISI Web of Science, Scielo, and EMBASE databases up to 29 April 2022. The search strategy performed in PubMed, which was adjusted for the other databases, is summarized in Table 1. The researchers manually patterned the list of references of each manuscript for the search of additional manuscripts. After the search, the papers were entered into Mendeley Desktop 1.17.11 software (Glyph & Cog, LLC, London, UK) to remove duplicates and then exported to the Rayyan web platform.

### 2.2. Study Selection

Two investigators (L.H. and R.B.) evaluated the abstracts and titles of all the articles using the blind mode on the Rayyan platform. Studies for full-text review were chosen based on the following eligibility criteria: (1) clinical studies assessing the clinical performance of direct or indirect restorations for treating tooth wear; (2) included a follow-up for at least 6 months; and (3) available in the English language. In vitro reports, case series, reviews, pilot studies, and case reports were omitted. Full versions of any possible reports were examined. Papers that had insufficient data in the abstract and title to offer a clear judgment were considered for full-text evaluation. The inter-examiner agreement was measured using the kappa coefficient. Any variations in the decision-making procedure in regard to the appropriateness of the accepted manuscripts was agreed and decided upon through the accord of a third author (C.E.C.-S.). Only texts that fulfilled all of the eligibility norms were incorporated for assessment.

### 2.3. Data Extraction

The information of interest from the papers selected was tabulated using a standardized sheet in a Microsoft Office Excel 2019 spreadsheet (Microsoft Corporation, Redmond, WA, USA). These data included type of clinical trial, number of the participants, reasons for tooth wear, restoration techniques used, follow-up, clinical criteria for evaluation, and main conclusion (Table 2).

### 2.4. Quality Assessment

The methodological quality of each included articles was individually evaluated by two reviewers (R.B. and L.H.) based on the Cochrane guidelines for the description of the subsequent parameters: selection bias (sequence generation and allocation concealment), performance and detection bias (blinding of operators or participants and personnel), bias due to incomplete data, reporting bias (selective reporting, unclear withdrawals, missing outcomes), and other bias (including industry sponsorship bias). A proposed judgement about the risk of bias arising from each domain was generated by an algorithm based on answers to the signaling questions. The aforementioned algorithm and the guidance on how to use it is available elsewhere [38]. Through risk of bias evaluation, any inconsistencies between the investigators were decided by a third reviewer (C.E.C.-S.).

## 3. Results

**Figure 1:**
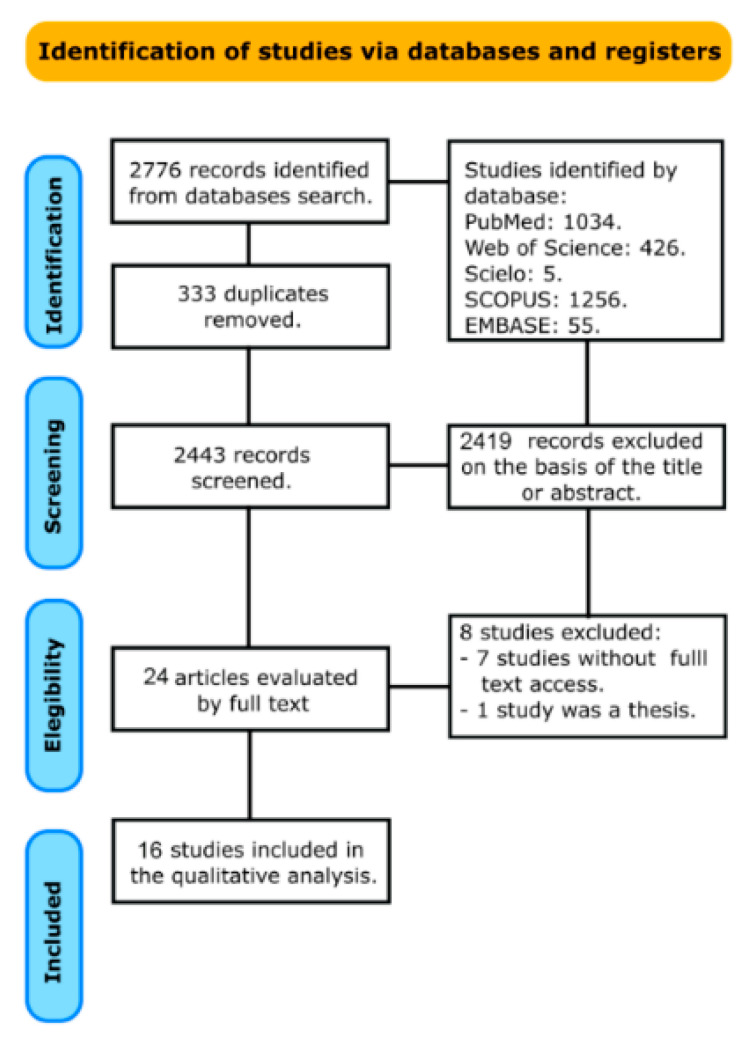
Flowchart according to the PRISMA statement.

A total of 2776 records were obtained from the search databases. After removing the duplicates, the total amount of manuscripts found was 2443 publications for the primary examination. Of these, 2419 papers were excluded after reviewing the titles and abstracts, leaving 24 articles to be selected for full-text review. The inter-examiner agreement was excellent (kappa coefficient = 0.87). Of these, eight studies were excluded [12,14,39,40,41,42,43,44]. Exclusion reasons are shown in the PRISMA flow diagram of the review (Figure 1), which resulted in a total of 16 articles for the qualitative analysis [3,13,16,18,20,21,23,45,46,47,48,49,50,51,52,53]. Considering the high heterogenicity of the studies included, a meta-analysis could not be performed.

**Table 2:** Qualitative analysis of the studies included.

Author and Year	Type of Clinical Trial	Number of Participants	Reason for Tooth Wear	Restoration Techniques Used	Follow-Up	Clinical Criteria for Evaluation	Main Conclusion
Bartlett 2006 [16]	Randomized clinical study	16 patients with severe tooth wear 13 controls without evidence of tooth wear	Mixture of bruxism and erosion	Direct or indirect microfilled resin composite restorations	3-year period	United States Public Health Service Evaluation System (USPHS) criteria	Using direct and indirect resin composites for fixing worn posterior teeth is contraindicated
Burian 2021 [49]	In-vivo study	Complex rehabilitations with deviations in vertical dimension of occlusion (VDO) 12 patients with severe tooth wear underwent prosthetic rehabilitation, restoring the VDO	Not described	Lithium disilicate ceramic (LS2) Experimental CAD/CAM polymer (COMP)	3-year period	Geomagic Qualify software (2 January 2012, Geomagic Inc., Morrisville, NC, USA) was used to compare resulting baseline and follow-up STL datasets.	LS2 presented less wear, yet tooth preparation was needed. Clinicians should balance well between required preparation invasiveness and long-term occlusal stability in patients with worn dentitions
Crins 2021 [48]	Randomized controlled trial	49 patients	Grinding/clenching and Gastro-Oesophageal Reflux Disease	Direct composite restorations (DRC) with micro-hybrid composite restorations (Clearfil AP-X, Kuraray) and nano-hybrid composite restorations (IPS Empress Direct, Ivoclar Vivadent) for buccal veneers indirect composite restorations with indirect palatal veneer restorations (Clearfil Estenia C&B, cemented with Panavia F, Kuraray)	3-year period	Functional (debond, fracture, adaptation, anatomy), Biological (caries, endodontic treatment) Esthetic conditions	Composite restorations showed superior behavior compared to the indirect composite restorations, when used in the molar region
Gresnigt 2019 [47]	Randomized split-mouth clinical trial	11 patients	Not described	48 indirect resin composite (Estenia) and ceramic laminate veneers (IPS Empress Esthetic)	10 years	USPHS criteria	Anterior ceramic laminate veneers might be favored over indirect composite laminate veneers
Hammoudi 2020 [46]	Randomized clinical trial	62 participants with extensive tooth wear	Mechanical (bruxism or engaged in vigorous labor or exercise), and chemical factors	713 lithium disilicate (LD) and translucent zirconia (TZ) crowns	65 months	USPHS criteria	The use of high-strength ceramic materials, as well as consistent adhesive bonding, are probably the key factors in the long-term success of ceramic crowns in participants with extensive tooth wear independent of the specific etiology
Hemmings 2000 [23]	Clinical study	16 patients	Not described	52 restorations composed of Durafill composite and Scotchbond Multipurpose dentine adhesive system 52 Herculite XRV composite and Optibond dentine bonding agent	30 months	Loss Fracture Marginal discoloration Loss of marginal integrity Noticeable wear Pain or sensitivity Endodontic failure Esthetic failure	Direct composite restorations may be a treatment option for localized anterior tooth wear
Katsoulis 2011 [45]	Observational study	42 patients	High daily consumption of tough and acidic food, reflux problems, bulimia combined with clenching and grinding	48 full prosthodontic rehabilitation	3 years	Complete oral examination Photos Functional and cast analysis General health conditions and behavioral aspects	The rehabilitation of partially edentulous patients with severe tooth wear is a complex task, and more information regarding treatment protocols, prosthetic indications and treatment outcome is needed
da Rocha Scalzer Lopes 2021 [50]	Retrospective study with cross-sectional design	43 individuals	Not described	112 single crowns	120 months	Analysis parameters of morphological variations in tooth wear are indicated	Ceramic systems can be considered as alternatives of restorative material, even in individuals with clinical features evocative of chronic tooth wear
Mehta 2021 [52]	Prospective trial	34 participants	Chemical (erosion) and mechanical wear (bruxism) signs	Direct restorations using a micro-hybrid (Clearfil AP-X; Kuraray, Japan) and a nanohybrid (IPS Empress Direct; Ivoclar Vivadent, Schaan, Liechtenstein) composite	1 month and 1-, 3-, and 5-years, post-treatment	Presence or absence of any symptoms of pain, difficulty with phonetics and/ or mastication, challenges with the adaption to the new VDO, or any TMJ-related concerns	Premolar restorations exposed lesser risks of failure compared to the molar restorations
Mehta 2021 (b) [51]	Prospective trial	34 participants	Chemical (erosion) and mechanical wear (bruxism) signs	Direct restorations using a micro-hybrid (Clearfil AP-X; Kuraray, Japan) and a nanohybrid (IPS Empress Direct; Ivoclar Vivadent, Schaan, Liechtenstein) composite	5.5 years	Presence or absence of any symptoms of pain, difficulty with phonetics and/ or mastication, challenges with the adaption to the new VDO, or any TMJ-related concerns	Molar restorations, posterior mandibular restorations and the anterior restorations requiring two further sessions for completion, were associated with significantly higher risks for failure
Milosevic 2016 [13]	Prospective trial	164 patients	Not described	Nano-particle hybrid composite material (Spectrum^®®^; Dentsply, Weybridge, UK)	8 years	Failure of the restoration	The assessed failure rate in the first year was 5.4%. Time to failure was significantly greater in older subjects and when a deficiency of posterior support was present. Bruxism and an increase in the Occlusal Vertical Dimension were not associated with failure
Oudkerk 2020 [18]	Prospective trial	7 patients	Chemical (erosion) and mechanical wear (bruxism)	PICN blocks (Vita Enamic HT, Vita Zahnfabrik, Germany; Ceramill Motion 2, Amann Girrbach)	One month, six months, 1 year and 2 years	World Dental Federation	PICN restorations displayed elevated survival and success rates after two years
Redman 2003 [20]	Retrospective	31 subjects	Primarily erosion, Primarily attrition, Combined erosion/attrition	Microfilled (Durafill), hybrid (Herculite—97 direct and 18 indirect) composites, and 73 indirect ‘ceromer’ (Artglass)	5 years	Modified United States Public Health Services criteria	Placement of resin-based composite restorations to treat localised anterior tooth wear has worthy short to medium term survival
Smales 2007 [3]	Retrospective	25 patients	Tooth grinding, gastric and dietary acids, and abrasive restorative materials	Resin-based composites (RBC), indirect ceramo-metal crowns (CMCs), and full gold crowns		Survival rate	RBCs usually failed from fractures, and CMCs from complete losses. RBC failures were usually replaced or repaired, while CMC failures often required root canal therapies or extractions
Taubóck 2021 [53]	Prospective trial	13 patients	Erosion-induced tooth wear and no signs of temporomandibular disorders	Microhybrid (first cohort; n = 59) or nanofilled (second cohort; n = 105) composite restorations	11 years	USPHS criteria	Direct composite restorations employed at an amplified vertical dimension of occlusion display suitable clinical long-term performance in patients presenting severe tooth wear
Vailati 2013 [21]	Prospective	12 patients	Presence of gastroesophageal reflux, excessive ingestion of acidic beverages	Direct and Indirect composite restorations (Miris, Coltène/Whaledent) and feldspathic ceramic veneers (Creation CC, Willi Geller International)	6 years	Modified United States Public Health Services criteria	Restoring compromised maxillary anterior teeth by means of veneers prevents excessive tooth structure removal and loss of tooth vitality

The features of the manuscripts included in this review are summarized in Table 2. This review identified randomized clinical trials and observational studies. The maximum follow-up observed in the included studies was 10 years. All studies included the rehabilitation of anterior or posterior teeth with extensive wear using both indirect and direct restorations. Tooth wear was described to be mainly due to the presence of gastroesophageal reflux, excessive ingestion of acidic beverages, tooth grinding, abrasive restorative materials, vigorous labor, or exercise.

Restoration materials included ceramo-metal crowns, full gold crowns, lithium disilicate ceramic, zirconia, polymer infiltrated ceramic networks, and resin composites. The last were used as both indirect and direct techniques. Most of the studies evaluated the survival rate of the restorations and the clinical characteristics using the USPHS Evaluation System criteria.

Studying the methodological quality assessment parameters, most of the studies included were counted as having a high risk of bias (Table 3), as most of them failed to avoid performance and detection bias, reporting bias, and other bias.

## 4. Discussion

This systematic review was focused towards examining the optimal treatment of tooth wear. This study included randomized clinical trials and observational studies for a maximum follow-up of 10 years. All the manuscripts included the rehabilitation of anterior and posterior teeth with extensive wear, using both indirect and direct restorations, with direct resin composite being the most common restorative treatment used. According to preceding reports, these materials seem to show acceptable fracture resistance and simulated wear rates [53,54,55,56]. In addition, they have shown suitable long-term achievement in other reports [53,55]. However, failure rates of approximately 10% were reported previously when comparing both materials after a mean follow-up of 30 months [23].

A previous systematic review performed in 2014 focused on the analysis of the steps that are recommended for treatment procedures when treating tooth wear, including diagnostic waxing, occlusal positioning, vertical dimension increase, restoration, and follow-up [8]. The present review is focused on the type of material (ceramic or resin-based) and the technique (direct or indirect) used for the treatment of tooth wear.

In addition, another important factor to be noted is that the etiology of the tooth wear for the studies included in this review could be divided mainly into two types: chemical and mechanical. Although none of the studies offered a comparison in the outcomes of the restorations according to the specific etiology, it should be recognized that different restorative materials do not have the same performance under different pH and mechanical challenges [57].

In the same way, it is worth mentioning that treating tooth wear in anterior teeth represents different challenges than treating tooth wear of posterior teeth. Further, different materials are indicated for both mouth regions, which is a comparison that is not presented in any of the manuscripts in this review.

Most of the researchers used the resin composites in both indirect and direct techniques. Restoring worn teeth by means of resin composites was advocated as a conservative and non-invasive procedure [3,23]. Further, resin composite restorations were inexpensive, provided an overall suitable esthetic appearance, and focused on additive instead of subtractive strategies [3,13,20,23]. However, most of the manuscripts on the restoration of worn teeth did not report long-term results of these restorative materials [20,23]. Preceding studies investigated the finding of resin composite and suggest that treating worn posterior teeth with these materials is contraindicated. This could be because of the brittle physical properties of the microfilled dental resin composites or the high loading forces on these restorations from either bruxing actions or increased vertical dimensions [16].

Another material found in this systematic review was ceramo-metal crowns. This material is seen as the standard of distinction for follow-up examination of clinical studies, and its performance was comparable to the metal-free systems (In-Ceram Alumina and feldspathic ceramic) [50,58]. With the development of adhesive dentistry, metal-free ceramic materials were established in response to the rising concern of biocompatibility and aesthetics [59]. Initially metal-free ceramics were characterized by conventional feldspathic ceramics and, subsequently, by reinforced ceramic systems [60].

Metal-free ceramic crowns display appropriate intrinsic characteristics such as color stability, compressive and abrasion resistance, chemical stability, coefficient of thermal expansion similar to that of the dental structures, radiopacity, and excellent potential to mimic the appearance of natural teeth as the chief materials in restorative dentistry [61]. Nevertheless, their inelastic feature can permit devastating fractures when the applied stresses touch the resistance of the material [62]. It should be emphasized that ceramic based materials such as feldspathic all-ceramic, metal-ceramic with a core in gold electropositive alloy, and In-Ceram Alumina can be considered as alternatives for treating individuals with tooth wear [50].

It is important to define the full gold crown used as an indirect single crown to treat advanced tooth wear in the elderly. A previous report examined the effect of this material and demonstrated lower proportions of failures when compared to direct resin-based composites and indirect ceramo-metal crowns. Most of these failures happened in anterior restorations, and this was observed at 10-year follow-up. Moreover, accumulative survival estimates were 62.0% for all direct restorations and 74.5% for all indirect restorations, including full gold crowns. This study showed no statistically noteworthy alteration between the survival of direct and indirect restorations and highlighted the importance of conducting large, long-term, controlled clinical trials to confirm these findings [3].

The present study demonstrated the use of lithium disilicate ceramic and zirconia crowns for treating patients with widespread tooth wear. One should bear in mind that metal–ceramic crowns are considered to be the standard treatment, as shown previously for crowns and fixed partial dentures [32]. However, this material has some drawbacks, including grayish discoloration at the gingival margin [63]. That is why materials with high strength, such as lithium disilicate and zirconia, have become widespread due to their appearance and biocompatibility [33,34]. Through a 6-year surveillance period, the use of both lithium disilicate and zirconia crowns showed promising survival rates of 99.7% when restoring extensive tooth wear. Normally, when 1 mm thick ceramic was inserted, bulk fracture did not happen (some zones in certain crowns were only 0.6 mm thick). Therefore, for patients with little remaining tooth tissue and extensive tooth wear, the use of minimally invasive high-strength ceramic crowns with cement seems to be helpful, regardless of the precise etiology. Nevertheless, zirconia crowns were rated by a blinded examiner as less esthetic than lithium disilicate crowns, knowing that no differences were found between both materials [46].

Knowing that tooth wear holds challenges for dental clinicians, novel solutions are needed for minimal invasive dentistry. This could be possible by using computer aided design (CAD)—computer aided manufacturer (CAM) technology. Polymer infiltrated ceramic with beneficial characters have been manufactured in the market [49]. These CAD—CAM polymers, launched under industrial standards, exhibit higher mechanical assets compared to those of direct polymers and have even been contemplated as a substitute to glass–ceramic [64,65,66]. Numerous benefits of CAD—CAM composites have been previously witnessed in diverse in vitro studies: high fatigue resistance, proper optical property, and an antagonistic friendly behavior [66,67]. Therefore, they were realized in distinctive fields of prosthetic dentistry [68,69]. Particularly in complex cases of worn dentition, the use of CAD—CAM-fabricated polymer allow for biomimetic methodologies and minimally invasive dentistry [68].

CAD—CAM polymers display important superior wear rates, with a mean vertical loss during the first year of 186 μm and 342 μm in premolar and molar regions, respectively. However, it should be noted that a full occlusal load had to be absorbed by these restorations. Consequently, use of an occlusal splint might be suggested for reducing the wear progression [49].

It should be highlighted that a 5-year recall showed no statistically significant differences between direct and indirect resin composites, and the authors recommended that these materials were preferable to those observed in other restorative materials [70]. Unfortunately, restoring severely worn posterior teeth involves alternatives such as more extensive prosthodontic techniques, comprising possibly elective endodontics and crown lengthening. Further research in this area is needed to investigate the optimal treatment of patients with tooth wear.

Most of the papers evaluated the survival rate of the restorations and the clinical characteristics using the United States Public Health Service Evaluation System criteria, as this criterion has gained considerable acceptability in clinical trials involving dental materials [21,53].

From this systematic review, clinical proof was evaluated with regard to compare the direct and indirect materials used in the treatment of worn teeth. The outcomes of this study should be carefully considered in clinical practice, as worn dentition could be caused by several factors, and defining the standard treatment option could not be done. Some of the studies lacked a sufficient time period, whereas other studies tested only indirect restorations without comparison to direct restorations. Thus, further inspection should focus on randomized controlled clinical trials, with the drive of reaching a better understanding of the performance of different materials in the clinical success of tooth wear in terms of novel materials and broad analysis. It is also recommended that research should focus on more consistent methods in an effort to lessen the heterogeneity among manuscripts on this topic and also to establish the ideal protocol for restoring tooth wear.

## 5. Conclusions

Contradictory discoveries were perceived concerning the type of restoration with better clinical performance. Considering the current literature available, there is no evidence in the superiority of any restoration technique to ensure the highest clinical performance for treating tooth wear. Further well designed randomized clinical trials are required in order to establish an optimal restoration technique protocol for the restoration of tooth wear.

## Figures and Tables

**Table 1:** Search strategy used in PubMed.

#1	Tooth Wear OR Tooth erosion OR Tooth attrition OR Dental Wear
#2	Restoration OR Direct Restoration OR Composite OR Resin Composite OR Composite Resin OR Dental Composite OR Resin Based Composite OR Composite Dental Resin OR Fillings OR Indirect Restoration OR Partial Restorations OR Posterior Partial Crowns OR Full-Coverage Restoration OR Ceramic OR Bonded OR Partial Preparations OR Indirect Bonded Restorations OR Porcelain OR Ceramic Veneer OR Overlay OR Inlay OR Onlay
#3	Clinical Trials OR Controlled Clinical Trial OR Retrospective Studies OR Randomized Controlled Trial OR Randomized Controlled Trials OR Prospective Clinical Trial OR Retrospective Study OR Prospective Studies OR Prospective Study OR Clinical Trial OR Randomized Clinical Trial OR Random Allocation OR Double-Blind Method OR Single-Blind Method OR Clinical Trial OR Clinical Trials OR Follow-up Studies OR Prospective Studies OR Cross-over Studies
#4	#1 and #2 and #3

**Table 3:** Risk of bias for clinical trials.

Study and Year	Selection Bias	Performance and Detection Bias	Bias Due to Incomplete Data	Reporting Bias	Other Bias
Bartlett 2006 [16]	Low risk	High risk	Low risk	High risk	High risk
Burian 2021 [49]	Low risk	High risk	High risk	High risk	High risk
Crins 2021 [48]	Low risk	Low risk	Low risk	High risk	High risk
Gresnigt 2019 [47]	Low risk	Low risk	Low risk	High risk	High risk
Hammoudi 2020 [46]	Low risk	Low risk	Low risk	Low risk	High risk
Hemmings 2000 [23]	Low risk	High risk	High risk	High risk	Low risk
Katsoulis 2011 [45]	High risk	High risk	High risk	High risk	Low risk
da Rocha Scalzer Lopes 2021 [50]	High risk	High risk	High risk	High risk	Low risk
Mehta 2021 [52]	Low risk	Low risk	High risk	High risk	Low risk
Mehta 2021 (b) [51]	Low risk	High risk	High risk	High risk	Low risk
Milosevic 2016 [13]	High risk	High risk	High risk	High risk	Low risk
Oudkerk 2020 [18]	High risk	High risk	Low risk	High risk	Low risk
Redman 2003 [20]	High risk	High risk	Low risk	High risk	Low risk
Smales 2007 [3]	High risk	High risk	High risk	High risk	High risk
Taubóck 2021 [53]	High risk	High risk	Low risk	High risk	High risk
Vailati 2013 [21]	High risk	High risk	Low risk	Low risk	High risk

## Data Availability

The data that support the findings of this study are available from the first author (L.H.) upon reasonable request.

## References

[1] Salas M., Nascimento G., Huysmans M., Demarco F. (2015). Estimated Prevalence of Erosive Tooth Wear in Permanent Teeth of Children and Adolescents: An Epidemiological Systematic Review and Meta-Regression Analysis. J. Dent..

[2] Van’t Spijker A., Rodriguez J.M., Kreulen C.M., Bronkhorst E.M., Bartlett D.W., Creugers N. (2009). Prevalence of Tooth Wear in Adults. Int. J. Prosthodont..

[3] Smales R.J., Berekally T.L. (2007). Long-Term Survival of Direct and Indirect Restorations Placed for the Treatment of Advanced Tooth Wear. Eur. J. Prosthodont. Restor. Dent..

[4] Shellis R.P., Addy M. (2014). The Interactions between Attrition, Abrasion and Erosion in Tooth Wear. Erosive Tooth Wear.

[5] Addy M., Shellis R. (2006). Interaction between Attrition, Abrasion and Erosion in Tooth Wear. Dent. Eros..

[6] Wetselaar P., Wetselaar-Glas M.J., Katzer L.D., Ahlers M.O. (2020). Diagnosing Tooth Wear, a New Taxonomy Based on the Revised Version of the Tooth Wear Evaluation System (TWES 2.0). J. Oral Rehabil..

[7] Li M.H., Bernabé E. (2016). Tooth Wear and Quality of Life among Adults in the United Kingdom. J. Dent..

[8] Muts E.-J., van Pelt H., Edelhoff D., Krejci I., Cune M. (2014). Tooth Wear: A Systematic Review of Treatment Options. J. Prosthet. Dent..

[9] Al-Khayatt A., Ray-Chaudhuri A., Poyser N., Briggs P., Porter R., Kelleher M., Eliyas S. (2013). Direct Composite Restorations for the Worn Mandibular Anterior Dentition: A 7-year Follow-up of a Prospective Randomised Controlled Split-mouth Clinical Trial. J. Oral Rehabil..

[10] Attin T., Filli T., Imfeld C., Schmidlin P.R. (2012). Composite Vertical Bite Reconstructions in Eroded Dentitions after 5·5 Years: A Case Series. J. Oral Rehabil..

[11] Hamburger J.T., Opdam N.J., Bronkhorst E.M., Kreulen C.M., Roeters J.J., Huysmans M.-C. (2011). Clinical Performance of Direct Composite Restorations for Treatment of Severe Tooth Wear. J. Adhes. Dent..

[12] Loomans B., Kreulen C., Huijs-Visser H., Sterenborg B., Bronkhorst E., Huysmans M., Opdam N. (2018). Clinical Performance of Full Rehabilitations with Direct Composite in Severe Tooth Wear Patients: 3.5 Years Results. J. Dent..

[13] Milosevic A., Burnside G. (2016). The Survival of Direct Composite Restorations in the Management of Severe Tooth Wear Including Attrition and Erosion: A Prospective 8-Year Study. J. Dent..

[14] Poyser N., Briggs P., Chana H., Kelleher M., Porter R., Patel M. (2007). The Evaluation of Direct Composite Restorations for the Worn Mandibular Anterior Dentition–Clinical Performance and Patient Satisfaction. J. Oral Rehabil..

[15] Edelhoff D., Güth J., Erdelt K., Brix O., Liebermann A. (2019). Clinical Performance of Occlusal Onlays Made of Lithium Disilicate Ceramic in Patients with Severe Tooth Wear up to 11 Years. Dent. Mater..

[16] Bartlett D., Sundaram G. (2006). An up to 3-Year Randomized Clinical Study Comparing Indirect and Direct Resin Composites Used to Restore Worn Posterior Teeth. Int. J. Prosthodont..

[17] Mainjot A.K.J. (2020). The One Step-No Prep Technique: A Straightforward and Minimally Invasive Approach for Full-mouth Rehabilitation of Worn Dentition Using Polymer-infiltrated Ceramic Network (PICN) CAD-CAM Prostheses. J. Esthet. Restor. Dent..

[18] Oudkerk J., Eldafrawy M., Bekaert S., Grenade C., Vanheusden A., Mainjot A. (2020). The One-Step No-Prep Approach for Full-Mouth Rehabilitation of Worn Dentition Using PICN CAD-CAM Restorations: 2-Yr Results of a Prospective Clinical Study. J. Dent..

[19] Mainjot A.K.J., Charavet C. (2020). Orthodontic-assisted One Step-no Prep Technique: A Straightforward and Minimally-invasive Approach for Localized Tooth Wear Treatment Using Polymer-infiltrated Ceramic Network CAD-CAM Prostheses. J. Esthet. Restor. Dent..

[20] Redman C., Hemmings K., Good J. (2003). The Survival and Clinical Performance of Resin–Based Composite Restorations Used to Treat Localised Anterior Tooth Wear. Br. Dent. J..

[21] Vailati F., Gruetter L., Belser U.C. (2013). Adhesively Restored Anterior Maxillary Dentitions Affected by Severe Erosion: Up to 6-Year Results of a Prospective Clinical Study. Eur. J. Esthet. Dent..

[22] Mesko M.E., Sarkis-Onofre R., Cenci M.S., Opdam N.J., Loomans B., Pereira-Cenci T. (2016). Rehabilitation of Severely Worn Teeth: A Systematic Review. J. Dent..

[23] Hemmings K.W., Darbar U.R., Vaughan S. (2000). Tooth Wear Treated with Direct Composite Restorations at an Increased Vertical Dimension: Results at 30 Months. J. Prosthet. Dent..

[24] Gow A.M., Hemmings K.W. (2002). The Treatment of Localised Anterior Tooth Wear with Indirect Artglass Restorations at an Increased Occlusal Vertical Dimension. Results after Two Years. Eur. J. Prosthodont. Restor. Dent..

[25] Elderton R. (1990). Clinical Studies Concerning Re-Restoration of Teeth. Adv. Dent. Res..

[26] Mehta S., Banerji S., Millar B., Suarez-Feito J.-M. (2012). Current Concepts on the Management of Tooth Wear: Part 2. Active Restorative Care 1: The Management of Localised Tooth Wear. Br. Dent. J..

[27] Lussi A., Hellwig E., Zero D., Jaeggi T. (2006). Erosive Tooth Wear: Diagnosis, Risk Factors and Prevention. Am. J. Dent..

[28] Loomans B., Opdam N., Attin T., Bartlett D., Edelhoff D., Frankenberger R., Benic G., Ramseyer S., Wetselaar P., Sterenborg B. (2017). Severe Tooth Wear: European Consensus Statement on Management Guidelines. J. Adhes. Dent..

[29] Van de Sande F., Opdam N., Da Rosa Rodolpho P., Correa M., Demarco F., Cenci M. (2013). Patient Risk Factors’ Influence on Survival of Posterior Composites. J. Dent. Res..

[30] Mehta S.B., Banerji S., Millar B.J., Suarez-Feito J.-M. (2012). Current Concepts on the Management of Tooth Wear: Part 4. An Overview of the Restorative Techniques and Dental Materials Commonly Applied for the Management of Tooth Wear. Br. Dent. J..

[31] Varma S., Preiskel A., Bartlett D. (2018). The Management of Tooth Wear with Crowns and Indirect Restorations. Br. Dent. J..

[32] Anusavice K.J. (2012). Standardizing Failure, Success, and Survival Decisions in Clinical Studies of Ceramic and Metal–Ceramic Fixed Dental Prostheses. Dent. Mater..

[33] Denry I., Kelly J.R. (2008). State of the Art of Zirconia for Dental Applications. Dent. Mater..

[34] Warreth A., Elkareimi Y. (2020). All-Ceramic Restorations: A Review of the Literature. Saudi Dent. J..

[35] Zhang Y., Lawn B. (2018). Novel Zirconia Materials in Dentistry. J. Dent. Res..

[36] Weigl P., Sander A., Wu Y., Felber R., Lauer H.-C., Rosentritt M. (2018). In-Vitro Performance and Fracture Strength of Thin Monolithic Zirconia Crowns. J. Adv. Prosthodont..

[37] Page M.J., McKenzie J.E., Bossuyt P.M., Boutron I., Hoffmann T.C., Mulrow C.D., Shamseer L., Tetzlaff J.M., Akl E.A., Brennan S.E. (2021). The PRISMA 2020 Statement: An Updated Guideline for Reporting Systematic Reviews. Int. J. Surg..

[38] Sterne J.A., Savović J., Page M.J., Elbers R.G., Blencowe N.S., Boutron I., Cates C.J., Cheng H.-Y., Corbett M.S., Eldridge S.M. (2019). RoB 2: A Revised Tool for Assessing Risk of Bias in Randomised Trials. BMJ.

[39] Aljawad A., Rees J.S. (2016). Retrospective Study of the Survival and Patient Satisfaction with Composite Dahl Restorations in the Management of Localised Anterior Tooth Wear. Eur. J. Prosthodont. Restor. Dent..

[40] Bartlett D., Sundaram G., Moazzez R. (2011). Trial of protective effect of fissure sealants, in vivo, on the palatal surfaces of anterior teeth, in patients suffering from erosion. J. Dent..

[41] Hamburger J.T. (2015). Treatment of Severe Tooth Wear: A Minimally Invasive Approach.

[42] Walls A. (1995). The Use of Adhesively Retained All-Porcelain Veneers during the Management of Fractured and Worn Anterior Teeth: Part 2. Clinical Results after 5 Years of Follow-Up. Br. Dent. J..

[43] Walls A. (1995). The Use of Adhesively Retained All-Porcelain Veneers during the Management of Fractured and Worn Anterior Teeth: Part 1. Clinical Technique. Br. Dent. J..

[44] Woodley N., Griffiths B., Hemmings K. (1996). Retrospective Audit of Patients with Advanced Toothwear Restored with Removable Partial Dentures. Eur. J. Prosthodont. Restor. Dent..

[45] Katsoulis J., Nikitovic S.G., Spreng S., Neuhaus K., Mericske-Stern R. (2011). Prosthetic Rehabilitation and Treatment Outcome of Partially Edentulous Patients with Severe Tooth Wear: 3-Years Results. J. Dent..

[46] Hammoudi W., Trulsson M., Svensson P., Smedberg J.-I. (2020). Long-Term Results of a Randomized Clinical Trial of 2 Types of Ceramic Crowns in Participants with Extensive Tooth Wear. J. Prosthet. Dent..

[47] Gresnigt M., Cune M., Jansen K., Van der Made S., Özcan M. (2019). Randomized Clinical Trial on Indirect Resin Composite and Ceramic Laminate Veneers: Up to 10-Year Findings. J. Dent..

[48] Crins L., Opdam N., Kreulen C., Bronkhorst E., Sterenborg B., Huysmans M., Loomans B. (2021). Randomized Controlled Trial on the Performance of Direct and Indirect Composite Restorations in Patients with Severe Tooth Wear. Dent. Mater..

[49] Burian G., Erdelt K., Schweiger J., Keul C., Edelhoff D., Güth J.-F. (2021). In-Vivo-Wear in Composite and Ceramic Full Mouth Rehabilitations over 3 Years. Sci. Rep..

[50] da Rocha Scalzer Lopes G., de Faria Viana A.A., Diniz V., de Matos J.D., Andrade V.C., Bottino M.A., Nishioka R.S., Chiarelli F.M., Feitosa A.C.R., Guerra S.M.G. (2021). Incidence of Fracture in Single Ceramic Crowns in Patients with Chronic Tooth Wear: A Clinical Follow-up. Int. J. Odontostomatol..

[51] Mehta S.B., Lima V.P., Bronkhorst E.M., Crins L., Bronkhorst H., Opdam N.J., Huysmans M.-C.D., Loomans B.A. (2021). Clinical Performance of Direct Composite Resin Restorations in a Full Mouth Rehabilitation for Patients with Severe Tooth Wear: 5.5-Year Results. J. Dent..

[52] Mehta S.B., Bronkhorst E.M., Lima V.P., Crins L., Bronkhorst H., Opdam N.J., Huysmans M.-C.D., Loomans B.A. (2021). The Effect of Pre-Treatment Levels of Tooth Wear and the Applied Increase in the Vertical Dimension of Occlusion (VDO) on the Survival of Direct Resin Composite Restorations. J. Dent..

[53] Tauböck T.T., Schmidlin P.R., Attin T. (2021). Vertical Bite Rehabilitation of Severely Worn Dentitions with Direct Composite Restorations: Clinical Performance up to 11 Years. J. Clin. Med..

[54] Alhadainy H.A., Abdalla A.I. (1996). 2-Year Clinical Evaluation of Dentin Bonding Systems. Am. J. Dent..

[55] Clelland N.L., Villarroel S.C., Knobloch L.A., Seghi R.R. (2003). Simulated Oral Wear of Packable Composites. Oper. Dent..

[56] Knobloch L.A., Kerby R.E., Seghi R., Berlin J.S., Clelland N. (2002). Fracture Toughness of Packable and Conventional Composite Materials. J. Prosthet. Dent..

[57] Lima V.P., Machado J.B., Zhang Y., Loomans B.A., Moraes R.R. (2022). Laboratory methods to simulate the mechanical degradation of resin composite restorations. Dent. Mater..

[58] Raposo L.H.A., Neiva N.A., da Silva G.R., Carlo H.L., da Mota A.S., do Prado C.J., Soares C.J. (2009). Ceramic Restoration Repair: Report of Two Cases. J. Appl. Oral Sci..

[59] Campos T., Ramos N., Machado J., Bottino M., Souza R., Melo R. (2016). A New Silica-Infiltrated Y-TZP Obtained by the Sol-Gel Method. J. Dent..

[60] de Matos J.D.M., Nakano L.J.N., Bottino M.A., de Jesus R.H., Maciel L.C. (2020). Current Considerations for Dental Ceramics and Their Respective Union Systems. Rev. Bras. Odontol..

[61] Erpenstein H., Borchard R., Kerschbaum T. (2000). Long-Term Clinical Results of Galvano-Ceramic and Glass-Ceramic Individual Crowns. J. Prosthet. Dent..

[62] Cehreli M.C., Kökat A.M., Ozpay C., Karasoy D., Akca K. (2011). A Randomized Controlled Clinical Trial of Feldspathic versus Glass-Infiltrated Alumina All-Ceramic Crowns: A 3-Year Follow-Up. Int. J. Prosthodont..

[63] Wall J.G., Cipra D.L. (1992). Alternative Crown Systems: Is the Metal-Ceramic Crown Always the Restoration of Choice?. Dent. Clin. N. Am..

[64] Mainjot A.K., Dupont N.M., Oudkerk J.C., Dewael T.Y., Sadoun M.J. (2016). From Artisanal to CAD-CAM Blocks: State of the Art of Indirect Composites. J. Dent. Res..

[65] Alt V., Hannig M., Wöstmann B., Balkenhol M. (2011). Fracture Strength of Temporary Fixed Partial Dentures: CAD/CAM versus Directly Fabricated Restorations. Dent. Mater..

[66] Stawarczyk B., Liebermann A., Eichberger M., Güth J.-F. (2016). Evaluation of Mechanical and Optical Behavior of Current Esthetic Dental Restorative CAD/CAM Composites. J. Mech. Behav. Biomed. Mater..

[67] Magne P., Schlichting L.H., Maia H.P., Baratieri L.N. (2010). In Vitro Fatigue Resistance of CAD/CAM Composite Resin and Ceramic Posterior Occlusal Veneers. J. Prosthet. Dent..

[68] Güth J., Edelhoff D., Goldberg J., Magne P. (2016). CAD/CAM Polymer vs Direct Composite Resin Core Buildups for Endodontically Treated Molars without Ferrule. Oper. Dent..

[69] Yilmaz B. (2018). CAD-CAM High-Density Polymer Implant-Supported Fixed Diagnostic Prostheses. J. Prosthet. Dent..

[70] Wassell R., Walls A., McCabe J. (2000). Direct Composite Inlays versus Conventional Composite Restorations: 5-Year Follow-Up. J. Dent..

